# Memorization Modulates the Lifespan of Event Files

**DOI:** 10.5334/joc.521

**Published:** 2026-09-28

**Authors:** Anna Scotts, Malte Möller, Ruyi Qiu, Iring Koch, Susanne Mayr

**Affiliations:** 1 Psychology and Human-Machine Interaction, University of Passau, Germany; 2 Department of Psychology, Hunan University of Chinese Medicine, Changsha, Hunan Province, China; 3 Psychology I: Cognitive and Experimental Psychology, RWTH Aachen University, Germany

**Keywords:** Event cognition, Short-term memory, Action and perception, Attention

## Abstract

The present study examined whether memorization influences retrieval of stimulus–response (S–R) representations, or event files. Repeating some but not all features of an event file impairs performance (partial repetition costs). Previous findings show smaller partial repetition costs after long than short intervals between formation and retrieval, suggesting that event file representations decay within a few seconds. Short-term memory processes such as rehearsal or attentional refreshing may mitigate this decay and preserve event files.

In this experiment (*N* = 223), participants performed an S1R1–S2R2 task and were either instructed to memorize S1 identity (memorization group) or received no such instruction (control group). Partial repetition costs were compared between short (500 ms) and long (5,000 ms) intervals between R1 and S2. The memorization group also completed a forced-choice S1-identity memory task after each trial.

In both groups, partial repetition costs were smaller after long than short R1-S2 intervals in response times and error rates. Partial repetition costs were also larger in the memorization group than in the control group across both intervals. In error rates, this group difference was modulated by interval length, with larger partial repetition costs following short intervals in the memorization group; a supplementary check showed this pattern was sensitive to a small number of trials, possibly reflecting reduced power (see Discussion). Exploratory analyses showed that better S1 memory performance was associated with slower event file decay. The findings provide evidence that memorization can mitigate event file decay, with implications for the interplay between binding, attention, and short-term memory.

## Introduction

In everyday life, it is often necessary to remember things or people after only a single encounter. For example, imagine meeting someone briefly who introduces themselves while you are in the middle of another conversation. Even though you hear the name only once, you may need to recall it shortly afterward when seeing that person again. With all the surrounding noise and competing information, it can easily be forgotten. However, keeping the person’s appearance together with their spoken name in mind just long enough can help to address the person correctly without asking again.

Features such as the person’s face (stimulus) and the spoken name (response) and their link are retained together and are assumed to be bound into a common episodic representation unit,^1^ often called an *event file* or a *stimulus-response (S-R) episode* (for overviews, see Hommel, 2004, and Binding and Retrieval Framework, BRAC, Frings et al., 2020). When any feature of an event file is re-encountered, the entire event file is assumed to be retrieved. Such retrieval can impair performance if the retrieved information partially mismatches either the subsequent stimulus or response. S–R binding and retrieval is typically measured using sequential paradigms, in which binding takes place in a so-called prime and event-file retrieval is assessed in a subsequent probe (Frings et al., 2020).

One well-established example of a sequential prime-probe presentation task is the S1R1-S2R2 paradigm (Hommel, 1998, 2005). In this paradigm, each trial typically begins with a cue (R1 cue) indicating the first response to be performed (R1). An initial stimulus (S1) is then presented, and participants execute R1 as instructed by the cue. Next, a second stimulus (S2) appears, requiring a second response (R2) according to a probe-response rule that remains constant throughout the experiment. Partial or complete feature overlap between S1/R1 and S2/R2 systematically influences R2 performance: repeating only stimulus- or response-related features between successive trials leads to longer response times and/or increased error rates as compared with full or no repetitions, also termed partial repetition costs (PRCs, Hommel, 2004).

Hommel’s theory of event coding characterizes event files as transient bindings that can be automatically retrieved, but it does not specify an explicit mechanism for sustained, attention-based maintenance in working memory or short-term memory (Hommel, 1998, 2004, 2009). In the BRAC framework (Frings et al., 2020) event files are conceptualized as memory-like entries comparable to Logan’s instances (Logan, 1988). More recent accounts propose that initial bindings vary in entry strength, partly depending on the activation of stimulus and response features through repetition and the time between repetitions. Only bindings that reach a certain strength threshold are assumed to be encoded in long-term memory; weaker bindings remain transient in short-term memory (Frings et al., 2024). Other accounts propose that event files are actively maintained in working memory via object-based attention, allowing recently integrated stimulus and response features to remain accessible (Lu et al., 2019). At present, it remains unclear how memory contributes to the formation and use of event files, and whether S–R bindings are solely short-lived, transient representations (Frings et al., 2024; Frings et al., 2020; Hommel, 1998), or can also become longer-lasting bindings. However, consensus is that memory plays a central role in S–R binding and retrieval. Yet, despite being recognized as a core aspect of event file research, direct empirical evidence on how memory shapes binding and retrieval and the lifespan of event files remains sparse.

Regarding the lifespan of event files, increasing the interval between event-file formation and later retrieval tends to reduce the impact of previous event files on performance, suggesting that event files are retrieved less strongly (or less frequently) over time (Frings et al., 2022; Hommel & Frings, 2020). In line with this, two studies varying several intervals between prime and probe trials have confirmed smaller PRCs with increasing intervals (Hommel & Frings, 2020). These results have been interpreted as evidence for decay rather than interference as the source of this decline: Hommel and Frings (2020) found no systematic effect of intervening stimuli or stimulus-response events on the drop in PRCs over time, and concluded that the reduction reflects the passage of time itself. It is worth noting that this decay is generally understood as a fading of activation in the bound codes rather than a dissolution of the link between them (Hommel & Colzato, 2004). In this view, an event file that has decayed may still be structurally intact, but harder to retrieve, rather than gone entirely.

This decay is not uniform across all binding types, however. How long a binding survives appears to depend on whether the bound feature was task-relevant. Frings (2011) and Frings et al. (2017) found that distractor-response bindings, formed from task-irrelevant features, survived a 500 ms interval but were no longer detectable after 2000 ms, whereas bindings involving task-relevant (target) features tend to persist over considerably longer intervals (Frings et al., 2022). Frings et al. (2022) directly compared target and distractor bindings within the same task and found that although target bindings were more durable overall, the two decayed at a comparable rate, differing mainly in their initial binding strength rather than in how quickly that strength was lost.

Based on this body of research, it has been discussed that the lifespan of event files is determined by attention, by shaping entry binding strength via attention allocation to relevant features and/or their attentional weighting in the binding process (Frings et al., 2020; Hommel, 2004). Hommel and Colzato (2004) investigated the effect of attention on the time course of event files and found that event files were more persistent across stimulus-onset asynchronies (SOAs) when the features of the first presented stimulus were attended than when they were unattended. In their second experiment, a visual version of the S1R1-S2R2 task (Hommel, 1998, 2005) was employed. The S1 and S2 were separated by an SOA ranging from 1,100 ms to 4,100 ms. In half of the six sessions, participants were instructed to pay attention to the S1—in other words, to memorize the features of the S1—as they had to respond to questions about one (of three) random S1 features after the trial. PRCs were largest at the shortest SOA and then decreased with increasing SOA levels in both response times and error rates, though they remained reliable even at the longest SOA. The effects of SOA on the PRCs were further modulated by the attention manipulation, but only in error rates, with event files being more persistent across SOAs when the S1 features were attended than when they were unattended. However, the authors concluded that attention was not necessary to observe the effect, and that the effect of attention and time on event files is rather limited, because PRCs were still present at the longest SOA in error rates.

Despite this progress, evidence for a mechanism affecting the event file longevity remains inconclusive, partly because previous studies focused on inducing decay to diminish memory traces (e.g. by added interfering stimuli or increasing intervals between presentations) or on attention processes that determine entry binding strength. Comparatively little consideration was given to factors that may support the persistence of event files once formed. In paradigms studying binding and retrieval, participants are typically not explicitly instructed to remember or memorize specific features (except see Hommel & Colzato, 2004).

Yet, processes such as rehearsal or attentional refreshing can preserve information in memory and counteract the loss of activation over time (Glavan & Houpt, 2019; Ricker et al., 2016). While both attentional refreshing and articulatory rehearsal can counteract memory loss, they operate via distinct mechanisms: attentional refreshing reactivates memory traces by directing attention toward them, whereas articulatory rehearsal maintains information through subvocal repetition via the phonological loop (Glavan & Houpt, 2019). Assuming that an event file is stored in short-term memory^2^ (Frings et al., 2024), manipulations designed to enhance short-term memory should prolong the persistence of these representations, leading to longer-lasting binding effects, operationalized here as larger PRCs. The present study aimed to test this possibility to clarify whether and how these memory processes affect event-file decay.

### Rationale of the Present Study

Following the idea that event files constitute short-term memory representations, manipulations that affect memory should influence the time course of transient event files. In this study, we incorporated elements from the narrative paradigm (Ezzyat & Davachi, 2011; Speer & Zacks, 2005) in a verbal version of the well-established S1R1-S2R2 task (Hommel, 1998) to increase the richness and concreteness of episodic representations. Robust binding and retrieval effects have been found with this task version in a previous study (Scotts et al., 2026).

We aimed to investigate whether instructing participants to memorize to later report the identity of an event-file element (S1) can affect PRCs. Beside the manipulation of the interval between event file formation (prime) and retrieval (probe), we manipulated whether participants were instructed to memorize information from the prime. Specifically, in the so-called *memorizing* condition, participants were asked to report the identity of the first stimulus (S1) after each trial. This requirement should promote rehearsal or attentional refreshing, which may counteract decay and help preserve information in memory (Glavan & Houpt, 2019; Ricker et al., 2016). Given that the stimuli in the present study were verbal, phonological rehearsal may be the more likely candidate mechanism underlying any memory benefit in the memorizing group.

PRCs were expected to be smaller following long R1-S2 intervals (i.e. 5,000 milliseconds) than short R1-S2 intervals (i.e. 500 milliseconds). In contrast to related work that manipulated S1-S2 SOAs (Hommel & Colzato, 2004; Hommel & Frings, 2020), we manipulated response-stimulus intervals (RSIs) between R1 and S2 to investigate event-file decay. RSIs appeared to be the most direct and cleanest implementation of a retention interval as it has been argued that the R1 response completes event-file formation and effectively closes the event file (Frings et al., 2020; Hommel, 2004). SOAs between S1 and S2, in contrast, conflate inter- and intra-individually varying R1 reaction time latencies and the actual retention interval. Consequently, RSIs may provide more direct control over the temporal distance between event file formation and retrieval than SOAs.

We therefore propose that the after-effects of stimulus-response binding decrease over time resulting in smaller PRCs after long RSI compared to short prime-probe RSI. Additionally, we assume that memory traces will decay more slowly in the memorizing group than those in the control group. More specifically, we expected larger PRCs in the memorizing than in the control group in the long interval condition, whereas there should be no difference or a smaller difference between the two groups in the short interval condition.

## Method

### Materials and Task

This experiment was part of a larger series of experiments in which we incorporated elements from the narrative paradigm (Ezzyat & Davachi, 2011; Speer & Zacks, 2005) in the well-established S1R1-S2R2 task (Hommel, 1998). Specifically, narrative texts were embedded in the task to create an impression of a story-telling role-play game. Each trial started with the presentation of a textual response cue (R1 Cue) followed by a prime (S1R1) and a probe (S2R2) presentation: For the R1 Cue, the German words for “Die Löwen: Rechts” or “Die Löwen: Links” (meaning “The lions: right” or “The lions: left” respectively) were presented for 2,000 ms, indicating a left or right response (R1) to perform at the time of the first stimulus (S1). The S1 was presented for up to 1,000 ms or until the R1 was performed. Although the S1 sentences varied (either the German words “Eine Birne erscheint” or “Eine Orange erscheint” meaning “A pear appears” or “An orange appears”, respectively), participants were instructed to respond solely with the response signalled by the cue, regardless of the identity of the S1. The relationship between the sentence presented as S1 and the response required in R1 was completely arbitrary. Responses were made using two spatially compatible keys (“f” for left and “j” for right) on a keyboard. Participants used the index fingers of each hand to press the keys.

The S2 probe sentences varied along the same dimensions (“pear” or “orange”) as the S1 in the prime, meaning S1 and S2, depending on the condition, could either match or mismatch. The response to the probe (R2) was not pre-signalled. Instead, the response to the S2 was governed by a rule (e.g., “pear” assigned to the left key and “orange” assigned to the right key or vice versa). The S2 was presented for up to 2,000 ms or until the R2 was performed. For the second stimulus (S2), the mapping between response keys and stimulus identities was counterbalanced across participants.

The following steps were performed to generate the trial set: First, all possible combinations between R1 cue (“left” vs. “right”), S1 (“orange” vs. “pear”), and S2 (“orange” vs. “pear”) were created, resulting in a total of eight trials. Among these trials, two unique trials belonged to each relation between stimulus and response: Stimuli and responses could either repeat or change between S1 and S2, as well as between R1 and R2, respectively. Thus, four trial types were produced (see Table 1). The trial set was combined with each of the two levels of the RSI factor (i.e., 500 ms, or 5,000 ms). The resulting 16 trials were presented four times creating the final set of 64 trials, which was presented in random order. Thus, the study length was kept to a minimum of 64 trials per participant to recruit participants via an online panel. During the RSI, three dots appeared successively with an interval of 100 ms between each dot and disappeared afterwards, see Figure 1.

**Table 1:** Trial types and examples for the stimuli and responses.


STIMULUS	RESPONSE			

	REPEATED		CHANGED	

**Repeated**	S1: A pear appears.	R1: j key	S1: A pear appears.	R1: f key

	S2: A pear appears.	R2: j key	S2: A pear appears.	R2: j key

**Changed**	S1: An orange appears.	R1: j key	S1: An orange appears.	R1: f key

	S2: A pear appears.	R2: j key	S2: A pear appears.	R2: j key


*Note*: S1 and S2 vary on the same dimension (“a pear appears” or “an orange appears”). R1 is governed by the R1Cue (“the lions: left” or “the lions: right”). The R2 is determined by the S2 rule (“a pear appears” or “an orange appears”).

**Figure 1:**
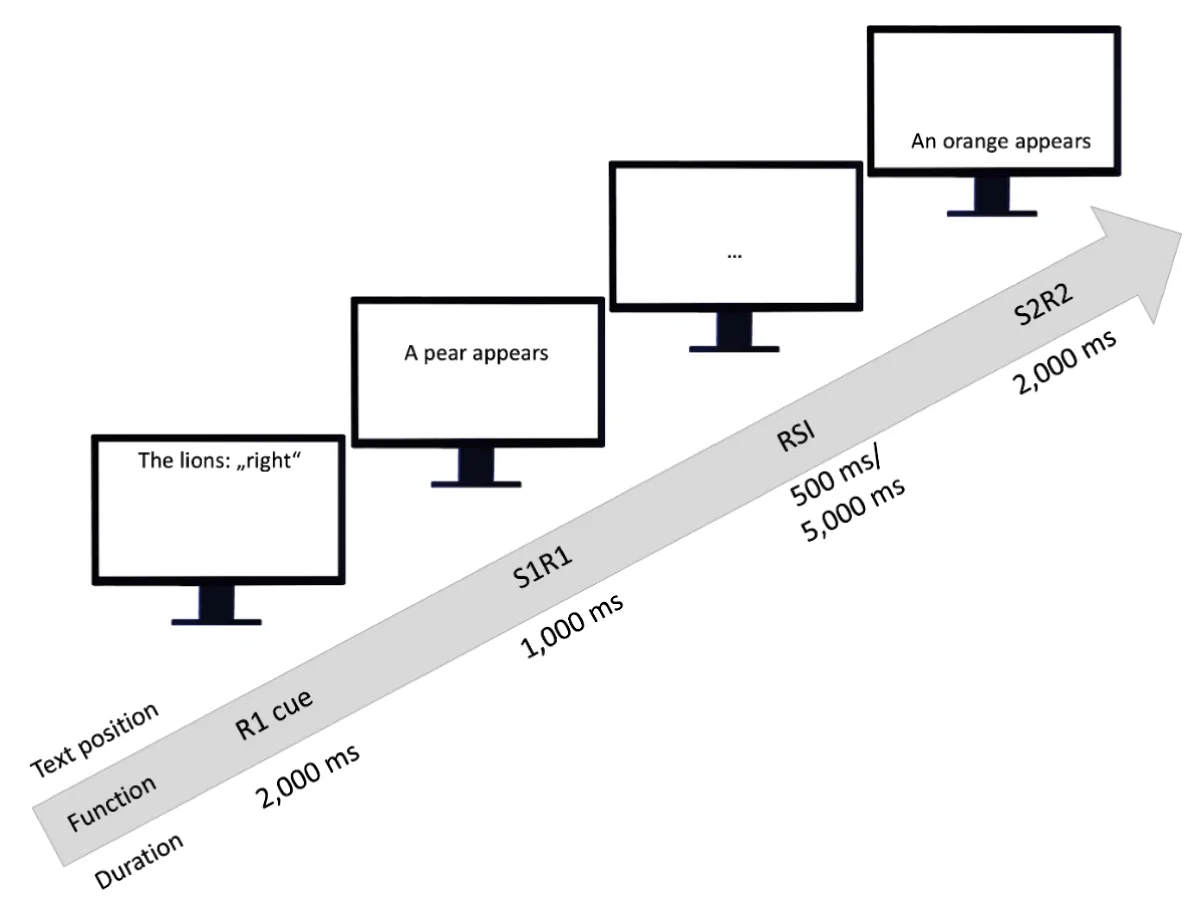
Example of a Trial sequence. *Note*. Across each trial, the text changed its on-screen position in a downward trajectory: the R1 cue appeared near the top of the screen, S1 and RSI placeholder were displayed at a mid-screen position, and S2 near the bottom. R1 was performed at the onset of S1, R2 was executed at the onset of S2.

### Procedure

The experiment was created using PsychoPy (Peirce et al., 2019) and was hosted on the Pavlovia platform (https://pavlovia.org). The experimental code was transformed into PsychoJS code. After giving informed consent via a mouse click, the narrative story embedding the S1R1 task was introduced: Participants were familiarised with instructions to take part in a magical tournament, called the “Trial of the Lions”, that involved passing a gate that was secured with two stone lions. The participants were informed that the lions would test their abilities in terms of speed, judgment, and depending on the group assignment, memory. Mirroring the procedure of an S1R1-S2R2 task, the lions would give them directional cues (R1 Cue) to test their speed (R1), show them the favourite fruits of the city’s royal couple (S2 and R2) to test the judgment abilities, and ask them questions about the order of presented fruits afterwards to test their memory in the memorizing group.

Participants underwent two practice blocks. In the first practice block (10 trials), only the R1 was performed. In the second practice block (12 trials), R1 and R2 were performed and, depending on the group assignment, memory questions for S1 were added after the R2. In the practice sessions, an accuracy of 50% of R1 and R2 respectively was needed to pass. If the participants failed in any training session, the experiment ended. Before entering the experiment phase, participants were told to respond as accurately and fast as possible. Each of the four experimental blocks contained 16 trials. After each trial, participants received audio-visual feedback about the correctness of the prime and probe responses (i.e., the written German words “Richtig” [“right!”] or “Falsch” [“wrong!”] and corresponding positive or negative spoken words such as “Ja” [“yes”], “Super” [“awesome”], or “Nein” [“no”]) followed by a 1,200 ms intertrial interval. For responses faster than 100 ms for R1 or R2 or slower than 1,000 ms for R1 and 2,000 ms for R2, as well as for responses with another than the two response keys, warning messages were presented, and the trial was aborted. After each trial, the participants in the memorizing group performed a forced choice binary memory check task without time limit (“Welche Frucht erschien zuerst? [“which fruit was shown first?”]) and answered via a mouse click on the button labelled “Orange” [“orange”] or “Birne” [“pear”]. After each block, participants were offered a rest of up to 10 minutes. Participants could resume the experiment at their own discretion. If the break exceeded 10 minutes, the program ended automatically. During block rests, participants received a summary of their accuracy during that block (for R1 and R2 separately in percent). If participants failed to respond more than seven times during a block (R1 and R2 together), the program ended automatically. The four experimental blocks took 13.64 (*SD* = 3.45) minutes on average to complete.

### Design and Analysis

Response times and error rates of R2 (given a correct R1) were of primary interest. The experiment comprises a 2 × 2 × 2 × 2 mixed design with RSI (500 ms vs. 5,000 ms), Stimulus (S1-S2, repeated vs. changed) and Response (R1-R2, repeated vs. changed) as within-subject variables and Memorization (memorizing vs. control) as between-subject variable, leading to 16 experimental conditions. Binding and retrieval effects are signified by the interaction between stimulus and response features, reflecting the integration and retrieval of event files. Partial repetition costs (reflecting the stimulus response interaction) for response times and error rates were calculated from the participants means in each condition as follows: (Stimulus_Changed_ Response_Repeated_ – Stimulus_Repeated_ Response_Repeated_) – (Stimulus _Changed_ Response_Changed_ – Stimulus_Repeated_ Response_Changed_) (e.g., Stoet & Hommel, 1999).

The analysis was carried out in three steps: First, a 2 × 2 × 2 × 2 mixed MANOVA was applied to response times and error rates of the R2. Second, to reduce complexity of the emerging data pattern, PRCs were reported instead of S-R binding effects (reflected by a significant Stimulus × Response interaction) in a 2 × 2 mixed MANOVA with the factors RSI (500 ms vs. 5,000 ms) and Memorization (memorizing vs. control). In case of a significant two-way interaction, repeated measures MANOVAs were applied to each RSI level. Third, to fully elucidate the pattern, PRCs for each RSI level were also tested against zero (via one-sided *t* tests).

### Participants

This experiment was conducted in accordance with the ethical guidelines of the German Association of Psychology (DGPs) and the Association of German Psychologists (BDP) and with the 1964 Declaration of Helsinki. The experiment was part of a research project which was approved by the University Research Ethics Committee of the University of Passau.^3^ In addition, the experiment was pre-registered at aspredicted.org (#190,425, https://aspredicted.org/dgbc-cmx3.pdf).

Sample size was estimated via power contours (Baker et al., 2021) using pilot data from two independent studies on the same topic (within *SD* = 161.00 ms, between *SD* = 133.04 ms and mean difference in PRCs, which represent the S-R interaction, PRC difference = 76.40 ms). Compared to traditional power analysis methods, which focus solely on sample size and do not consider the number of trials per participant, power contours simultaneously optimize both the number of participants and the number of trials per participant, with the relative magnitude of within- versus between-participant variance determining what the critical trial number is for achieving adequate power. For desired power of 95% and 32 trials per Response Stimulus Interval (RSI) factor level, data had to be collected from 210 participants. Although this resulted in only four trials per condition, power was ensured by recruiting a large sample consistent with the recommendation that power can be improved by increasing either the number of trials or the number of participants (Miller, 2024). A total of 232 participants were recruited via an online panel service (Bilendi, https://www.bilendi.de/) (116 participants per group). Seven participants were excluded due to excessive error rates in the first response (R1, 3 participants) or the second response (R2, 4 participants) amounting to at least 40% overall error rates in R1 or R2 (given a valid R1 response) (as compared with an average of 8.27% in R1 and 6.73% in R2), suggesting either their inability or unwillingness to follow the instruction. Additionally, one participant was excluded due to a lack of correct responses in one condition.^4^ Finally, one participant was excluded due to excessive time to complete the experiment (323 minutes compared to average of 20 minutes).^5^ No one was excluded due to excessive error rate in memory checks for S1 within the memorizing group (*M* = 5.14%, *SD* = 5.22%). In the final sample, 109 participants remained in the memorizing group and 114 in the control group, respectively. The total sample of *N* = 223 (176 women, 46 men, 1 diverse), ranged in age from 18 to 30 years (*M* = 24.63, *SD* = 3.18).

## Results

### Data Pre-processing

Trials with an invalid R1 were excluded (8.27%). This included trials with responses faster than 100 ms (anticipations, 0.09%), with R1 latencies longer than 1,000 ms (6.11%) and incorrect responses (2.07%). From trials given a correct R1, anticipated R2 responses (0.32%), and trials with R2 latencies longer than 2,000 ms (0.32%) were treated as invalid and were excluded from hypotheses-relevant analyses. For response times, trials with incorrect R2 were excluded (6.09%). Out of the 8 trials per cell, on average 7.40 trials for R1 and 7.08 trials for R2 were used for response time analysis.

### Response times: *Partial Repetition Costs*

For descriptive statistics, please see Table 2. With respect to S-R binding and retrieval effects (i.e., PRCs) in response times, there was a significant effect of Memorization, *F*(1, 221) = 28.71, *p* < .001, η_p_^2^ = .11, indicating larger PRCs for the memorizing group (*M_PRC_* = 117.07, *SD_PRC_* = 153.66) compared to the control group (*M_PRC_* = 42.07 ms, *SD_PRC_* = 130.65 ms). There was also an effect for RSI, *F*(1, 221) = 13.64, *p* < .001, η_p_^2^ = .06, confirming generally larger PRCs after the 500 ms RSI (*M_PRC_* = 102.03 ms, *SD_PRC_* = 166.42 ms) as compared with the 5,000 ms RSI (*M_PRC_* = 55.42 ms, *SD_PRC_* = 120.72 ms). Effects of RSI were not modulated by Memorization (the interaction of Memorization and RSI was not significant, *F*(1, 221) = 0.30, *p* = .58, η_p_^2^ < .01). PRCs were present in both groups in both RSIs (*t*-test against zero, all *p’s* < .05), see Figure 2. A full overview of the MANOVA results for S-R binding and retrieval, is provided in the Supplementary Material in Table S1.

**Table 2:** Descriptive statistics (means, standard deviations in parentheses) for response times in milliseconds as a function of Stimulus, Response, RSI, and Memorization as well as PRCs by RSI and Memorization.


MEMORIZATION	STIMULUS	RESPONSE				PRCs	
	
		500 ms-RSI		5,000 ms-RSI		500 ms-RSI	5,000 ms-RSI
	
		REPEATED	CHANGED	REPEATED	CHANGED		

Memorizing	Changed	771 (177)	707 (142)	710 (147)	665 (133)	144 (178)	90 (120)

	Repeated	676 (136)	755 (167)	651 (139)	696 (142)		

Control	Changed	707 (117)	674 (125)	651 (115)	638 (106)	62 (144)	22 (113)

	Repeated	668 (131)	698 (129)	639 (110)	648 (104)		


*Note*. RSI = Response stimulus interval. PRCs = Partial repetition costs (reflecting the stimulus response interaction, operationalized as the performance cost when either the stimulus repeats while the response changes, or the response repeats while the stimulus changes) for response times were calculated from the participants means in each condition as follows: (Stimulus_Changed_ Response_Repeated_ – Stimulus_Repeated_ Response_Repeated_) – (Stimulus_Changed_ Response_Changed_ – Stimulus_Repeated_ Response_Changed_). Values are rounded to the nearest ms.

**Figure 2:**
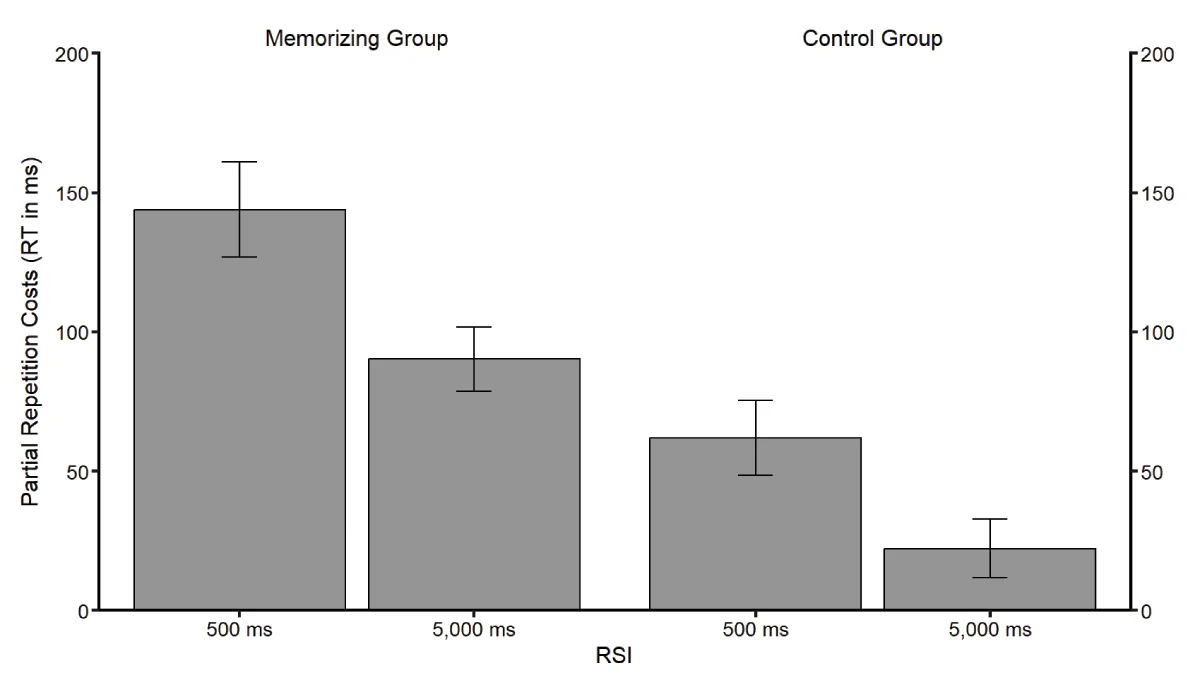
PRCs for response times in milliseconds as a function of Memorization and RSI. *Note*. The terms “RT” and “RSI” refer to response time and response-stimulus interval, respectively. Error bars represent the standard errors of the means.

### Error Rates: *Partial Repetition Costs*

For descriptive statistics for error rates please see Table 3. PRCs, reflecting S-R binding and retrieval effects, for error rates were generally larger in the memorizing group (*M_PRC_* = 12.31%, *SD_PRC_* = 21.33%) compared to the control group (*M_PRC_* = 6.33%, *SD_PRC_* = 19.19%), which can be seen in the effect of Memorization, *F*(1, 221) = 9.87, *p* < .01, η_p_^2^ = .04. PRCs were also generally larger after the 500 ms RSI (*M_PRC_* = 15.18%, *SD_PRC_* = 23.22%) as compared with the 5,000 ms RSIs (*M_PRC_* = 3.33%, *SD_PRC_* = 15.15%), as indicated by the effect of RSI, *F*(1, 221) = 45.52, *p* < .001, η_p_^2^ = .17. PRCs were smaller in both groups after the 5,000 ms RSI compared to the 500 ms RSI (memorizing group *F*(1,108) = 44.71, *p* < .001, η_p_^2^ = .29, control group *F*(1, 113) = 9.92, *p* < .01, η_p_^2^ = .08). Unlike in response times, the effect of RSI was modulated by Memorization (interaction between Memorization and RSI: *F*(1, 221) = 4.50, *p* < .05, η_p_^2^ = .02) but this interaction was mainly driven by the difference in the 500 ms RSI between the groups, *F*(1, 221) = 10.128, *p* < .01, η_p_^2^ = .04; there was no significant difference between the groups for the 5,000 ms RSI, *F*(1, 221) = 1.23, *p* = .27, η_p_^2^ < .01. PRCs appear to be present in both groups in both RSIs (*t* test against zero, all *p’s* < .001, although there was only a tendency, *t*(113) = 1.49, *p* = .07, for the 5,000 ms RSI condition in the control group with no instruction to memorize), see Figure 3. A full overview of the MANOVA results for S-R Binding and Retrieval, is provided in the Supplementary Material in Table S2.

**Table 3:** Descriptive statistics (means, standard deviations in parentheses) for error rates in percent as a function of Stimulus, Response, RSI, and Memorization as well as PRCs by RSI and Memorization.


MEMORIZATION	STIMULUS	RESPONSE				PRCs	
	
		500 ms-RSI		5,000 ms-RSI		500 ms-RSI	5,000 ms-RSI
	
		REPEATED	CHANGED	REPEATED	CHANGED		

Memorizing	Changed	13(16)	2 (6)	6 (11)	4 (7)	20 (24)	4 (14)

	Repeated	3 (6)	12 (15)	2 (6)	2 (6)		

Control	Changed	10 (14)	4 (7)	7 (11)	5 (10)	10 (21)	2 (16)

	Repeated	6 (9)	9 (13)	4 (7)	4 (8)		


*Note*. RSI = Response stimulus interval. PRCs = Partial repetition costs (reflecting the stimulus response interaction, operationalized as the performance cost when either the stimulus repeats while the response changes, or the response repeats while the stimulus changes) for error rates were calculated from the participants means in each condition as follows: (Stimulus_Changed_ Response_Repeated_ – Stimulus_Repeated_ Response_Repeated_) – (Stimulus_Changed_ Response_Changed_ – Stimulus_Repeated_ Response_Changed_). Values are rounded to the nearest percentage.

**Figure 3:**
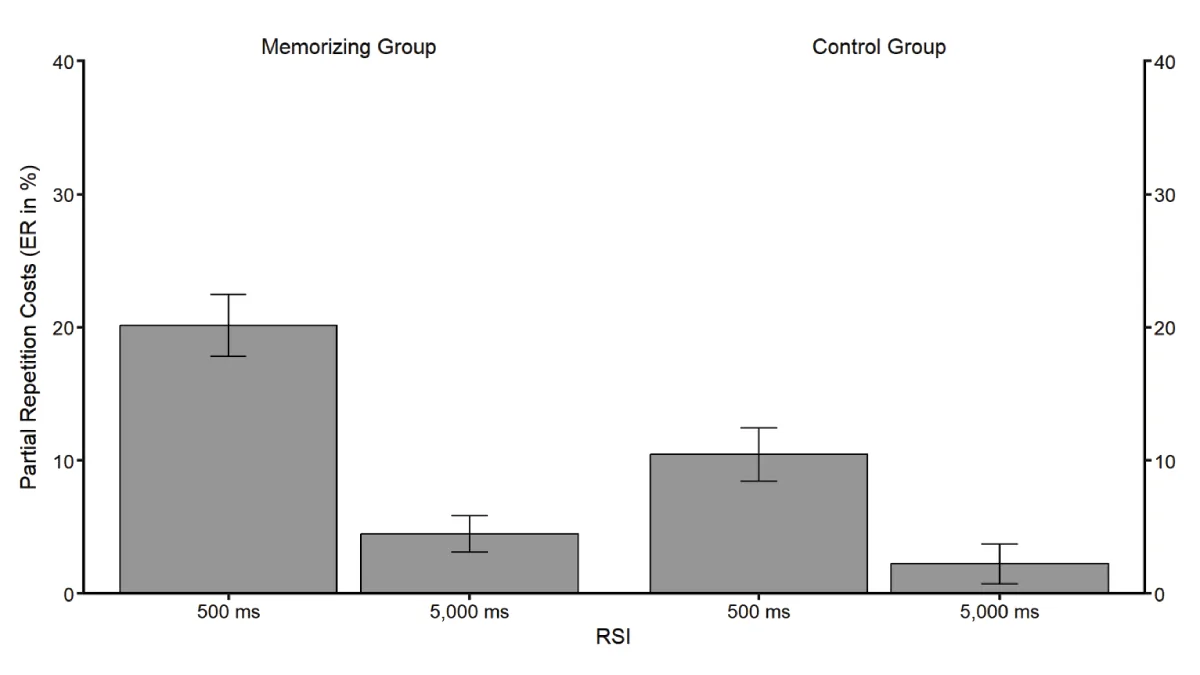
PRCs for error rates in percent as a function of Memorization and RSI. *Note*. The terms “ER” and “RSI” refer to error rates and response-stimulus interval, respectively. Error bars represent the standard errors of the means.

### Memory Performance in the Memory Check Trials

Because the memory check used a two-alternative forced-choice format (pear vs. orange), overall discriminability demands were low, and accuracy was correspondingly high *(M* = 95.86% *SD* = 5.60% at the 500 ms-RSI and *M* = 93.88% *SD* = 6.51% at the 5,000 ms-RSI). Memory-check accuracy increased significantly across the session rather than declining, *b* = 0.025, *SE* = 0.0007, *z* = 35.79, *p* < .001 (mixed-effects logistic regression predicting trial accuracy from centered trial number, with a random intercept for participant). Adding an RSI × trial-number interaction significantly improved model fit, χ²(2) = 12.03, *p* = .002, but the interaction term itself was not reliable, *b* = –0.002, *SE* = 0.0065, *z* = –0.35, *p* = .729; the two RSI conditions differed in overall accuracy (higher for 500 ms-RSI, *b* = 0.39, *SE* = 0.12, *z* = 3.17, *p* = .002) but not in how accuracy changed across the session (see Supplementary Figures S3–S5).

### Post-hoc Analysis: Error Rate in Memory Checks and PRCs

The S1 memory check error rate in the memorizing group was positively correlated with the difference in PRCs between short and long RSIs in response times, *r* = .35, *t*(107) = 3.90, *p* < .001 (as for the control group there were no S1 memory checks, a corresponding analysis was therefore not possible). More specifically, the more errors participants committed in the S1 memory check questions, the more detrimental was the long RSI for the PRCs, suggesting that the decay rate of event files as the RSI increases was faster when error rates were higher for memorizing the S1. This suggests that the accessibility of stimulus identity information and of event files is related to each other. For error rates, the corresponding correlation was not significant, *r* = .08, *t*(107) = 0.88, *p* = .382, suggesting that the relationship between memory performance and PRC stability observed in response times did not extend to error rates.

## Discussion

In this experiment, we investigated whether the instruction to memorize the identity of an event-file element (S1) affects the persistence of event files, indexed by PRCs. Assuming that event files are stored in short-term memory, maintaining the memory trace of an event file should influence its life span. In other words, we proposed a memory trace will last longer in the memorizing group as compared with the control group. Based on previous work (Hommel & Frings, 2020), we assumed smaller PRCs after long as compared with short prime-probe RSI. Additionally, we expected larger PRCs for the memorizing group as compared with the control group after the long RSI, whereas we expected no difference or a smaller difference between the two groups after the short RSI.

As predicted and in line with previous work (Hommel & Frings, 2020), overall PRCs were smaller after long (i.e. 5,000 ms) as compared with short (i.e., 500 ms) R1-S2 RSIs in response times and error rates, indicating that retrieval of event files is reduced after long RSIs. Previously, this reduction in PRCs over longer intervals has been attributed to decay (Hommel & Frings, 2020), consistent with an account in which the link between bound codes persists but becomes harder to reactivate over time. Furthermore, PRCs in response times and error rates in our study were also generally larger when S1 had to be explicitly memorized compared to when no instruction was given to memorize it. In response times, this effect of Memorization was not modulated by time (RSI): the instruction to memorize improved the retrieval of event files at all levels of the RSI factor. In error rates, PRCs differed between the groups after short RSIs but not after long RSIs.

This dissociation between response times and error rates is worth being addressed. In response times, the memorizing group showed larger PRCs than the control group at both RSIs equally; in error rates, this group difference was present at the short RSI but not the long RSI. This is not a classic speed-accuracy trade-off, which would predict opposite directions across the two measures. A more likely explanation is that the two measures differ in their sensitivity. Measure-specific sensitivity has also been reported in other event-file literature: Hommel and Colzato (2004) reported several effects, including the impact of stimulus location repetition, that were significant in one measure but not the other, and interpreted this as reflecting differences in how sensitive each measure is to the underlying process rather than evidence for two dissociable effects. The same explanation likely applies here, though we cannot rule out that the RT effect is present but underpowered to detect the interaction specifically, given that the main effect of Memorization in response times was reliable.

To assess whether the RSI × Memorization interaction in error rates was driven by trials with an incorrect S1 memory check, we re-ran the primary analysis excluding these trials (4.33% of trials in the memorizing group).^6^ This interaction was reduced to a non-significant trend under the restricted sample, while the corresponding interaction in response times was essentially unchanged (see Supplementary Table S4). This restricted analysis has lower power to detect a true effect of the same size, as PRC variance in the memorization group was significantly larger than in the control group (see Supplementary Table S4), making it harder to detect an interaction effect. We therefore do not interpret this shift as evidence that the error-rate interaction is an artefact.

We also attempted to compare PRCs directly between memory-check-correct and memory-check-incorrect trials within the memorizing group. This comparison proved uninterpretable: computing a within-participant PRC for the incorrect-trial subset requires at least one incorrect-memory-check trial in each of the four Stimulus × Response conditions, at both RSIs (eight cells in total) and only a single participant met this requirement (see Supplementary Table S5). We therefore do not report this comparison.

We attribute this shift toward non-significance in the error-rate interaction to reduced power rather than to a disappearance of the underlying effect. This does not undermine the broader pattern: the overall tendency toward larger PRCs in the memorizing group held across both response times and error rates, and across the full and restricted samples alike. Rather than slowing the decay of an existing memory trace, the instruction to memorize may instead have strengthened the initial binding of stimulus and response features. This interpretation is consistent with recent accounts proposing that entry binding strength determines the retrievability of event files over time, such that stronger bindings are retrieved more reliably regardless of the elapsed interval (Frings et al., 2024). Attention allocated to stimulus features during encoding is one factor proposed to increase entry binding strength (Frings et al., 2024), and the requirement to memorize the S1 identity may have directed attention toward the S1 in a way that enhanced this initial binding in addition to supporting active maintenance over the delay.

Hommel and Colzato (2004) argued that binding does not depend on attention or available attentional resources per se, but is instead governed by the current task goal: once a goal makes a particular feature dimension relevant, that relevance can be held in working memory and lend top-down support to the corresponding feature, after which integration proceeds automatically without further effort. Our memorization instruction can be understood as one way of establishing such a task goal, making S1 identity relevant in a way it was not for the control group, which is consistent with Hommel’s and Colzato’s account. Relatedly, event files were more persistent across SOAs when attention was directed to S1 features, in error rates, in Hommel and Colzato’s (2004) own data. However, Hommel’s and Colzato’s conclusion concerned whether attention is necessary for binding to occur, whereas our findings speak to whether a memorization instruction can enhance the persistence of an already-formed event file. These are compatible claims: our control group, which showed reliable PRCs without any memorization instruction, replicated Hommel’s and Colzato’s finding that binding does not require attention. The data from the memorizing group showed, in addition, that explicitly instructing participants to retain S1 can strengthen this effect further.

When comparing the two studies, several further differences should be noted. In the present study, only a single verbal stimulus (i.e. identity of the object) had to be memorized instead of three features (colour, shape, location) in the study of Hommel and Colzato (2004). Using a single stimulus feature likely facilitated phonological rehearsal and may have strengthened the influence of memory on event-file representations. With respect to the interval type, the temporal distance between S1 and S2 is shorter when SOAs are used, as in Hommel and Colzato (2004), than when the same intervals are employed as RSIs, as in the present study, because the participants’ response time is added to the interval in the latter case. Longer intervals may increase the risk of attenuating binding and retrieval effects (Hommel & Colzato, 2004; Hommel & Frings, 2020). Nevertheless, partial repetition costs, the index of binding and retrieval effects, were observed even following the long RSIs (5,000 ms) in our study, despite the longer elapsed time between S1 and S2 (up to 6,000 ms) compared to long SOAs (4,000 ms) in Hommel and Colzato (2004), as in absolute terms, the type of interval (RSI vs. SOA) does make a difference for the time elapsed between S1 and S2. By controlling the interval between response and subsequent stimulus, rather than between stimuli, the interval manipulation targets stimulus–response integration more directly and is in line with the common assumption that the response completes and effectively closes an event file (Frings et al., 2020; Hommel, 2004). This indicates that using RSIs instead of SOAs does not compromise and may even strengthen the experimental design and event-file formation. Finally, with *N* = 223, the present study is considerably larger than Hommel and Colzato (2004), *N* = 16, providing substantially greater statistical power to detect modulations of attention on the time course of PRCs, keeping in mind that between-subject designs are statistically less sensitive than within-subject designs.

The idea of the manipulation was to strengthen the representation of S1 to promote the event file binding between S1 and R1. Alternatively, due to the narrative setting, it could be argued that the memorization instruction did not selectively boost S1’s representation. Instead, the narrative might have encouraged participants to integrate S1, R1, and S2 into a single episode. If so, the larger PRCs in the memorizing group could reflect a more comprehensive event file spanning both trial segments rather than a more durable S1 trace specifically.^7^ While it is more parsimonious that fostering the representation of S1 promotes the longevity of the S1-R1 event file, the current experiment cannot rule out that a broader episode was formed and boosted by the memorization.

One limitation of our study is that our design does not disentangle binding from retrieval. We have interpreted the larger PRCs in the memorizing group as reflecting a stronger initial binding between S1 and R1, but the memorization instruction could equally have affected how that binding is retrieved at S2, rather than how strongly it was formed at encoding. Because participants in the memorizing group knew a memory check would follow, they may have engaged in a more effortful retrieval search at S2, independent of any difference in the strength of the underlying S1-R1 binding itself. Our design cannot separate a stronger entry binding from a more effective retrieval process, and the present findings should be read as speaking to the combined outcome of binding and retrieval rather than to either process in isolation.

Within the memorizing group, the better participants performed in the S1 memory task, the less their PRCs decreased as the RSIs became longer. This association between S1 memory performance and the stability of PRCs points to the role of memory for the retrievability of event files (with the corresponding finding being robust to the exclusion of outliers; see Supplementary Figures S1–S2 and Table S3). Memory accuracy was close to ceiling in this sample (*M* = 94.86%). Restricted range of this kind would typically be expected to attenuate rather than inflate an observed correlation. That the association remained detectable, and survived the exclusion of outliers, therefore supports a true underlying relationship rather than an artefact of restricted range. We nonetheless note that with most participants clustered at the high end of the accuracy distribution, the shape of this relationship and whether it holds with the same form may look different in a task placing greater demands on memory.

Notably, accuracy did not simply sit at a stable ceiling throughout the session; it improved significantly across trials (see Results). This pattern is inconsistent with a proactive-interference account, which predicts declining accuracy across trials, and is more consistent with a practice effect in the memory-check procedure itself. This further supports treating the observed ceiling as a property of the task’s low discriminability demands rather than as evidence that memory performance was uniformly at its best throughout, and reinforces the caution urged above in interpreting the correlation with PRC stability. The short-RSI accuracy advantage reported above is consistent with some forgetting over the longer retention interval. Although the introduction framed our predictions primarily in terms of spontaneous decay, the present findings suggest that both the initial strength of binding and the active maintenance of memory traces over time contribute to event file retrievability. These two processes are not mutually exclusive: a stronger binding may prolong the time at which an event file is accessible, while active maintenance may further counteract loss of activation during the retention interval. What form this active maintenance takes, attentional refreshing, phonological rehearsal, or a combination, remains an open question.

## Conclusion

The present research shows how memory processes contribute to the strength and persistence of event files. Explicitly instructing participants to memorize stimulus information increased partial repetition costs regardless of the interval between events, suggesting that memory can strengthen event-file binding at encoding rather than merely slowing its decay. Individual differences in memory performance showed a parallel pattern, further clarifying how memory contributes to how long these bindings last. These results position memory as an active contributor to event-file formation, rather than a purely automatic, perception-driven process.

## Transparency and Openness

We comply with the TOP guidelines. The study was pre-registered at aspredicted.org (#190425, https://aspredicted.org/dgbc-cmx3.pdf). Data were collected in the year 2024.

## Additional File

The additional file for this article can be found as follows:

10.5334/joc.521.s1 Supplementary Material. Supplementary tables and figures reporting: the full 2×2×2×2 mixed MANOVA on response times and error rates (Tables S1–S2); a robustness check of the memory-check-error/PRC correlation against outliers (Figures S1–S2, Table S3); memory-check accuracy across the course of the experiment (Figures S3–S5); a robustness check of the error-rate Memorization × RSI interaction excluding incorrect memory-check trials (Table S4); and a comparison of PRCs following correct versus incorrect memory-check trials (Table S5).

## Data Availability

The data and the code to reproduce the analyses are available at: Data: https://doi.org/10.23668/psycharchives.22512 Code: https://doi.org/10.23668/psycharchives.22513

## Footnotes

Yet, there is an ongoing discussion of how the stimulus and response features are precisely retained. Another view suggests that features and their links are stored separately (Verwey, 2024).

This process presumably relies on short-term memory, because the sensory register as an alternative location for (very) short-term storage has been argued to be modality-specific and therefore cannot integrate perceptual and motor representations.

This specific experiment was not described in the ethics application; however, it was very similar in design to those approved and the ethics application already announced additional experiments with minor variations, which was approved. The memorizing manipulation was not described in the application for approval by the ethics committee. In addition, the current experiment was significantly shorter (only 64 trials) than the experiments described and approved in the application (192+ trials). Data collection and payment of participants was carried out via an online panel (Bilendi, also not described in the ethics application).

This is a deviation from the pre-registration.

This is a deviation from the pre-registration.

We thank Carina Giesen for suggesting this analysis.

We thank Bernhard Hommel for suggesting this alternative interpretation.
