# Supplementary material for: Memorization Modulates the Lifespan of Event Files
Source: J Cogn. 2026 Sep 28;9(1):49. doi: 10.5334/joc.521 (PMC13629531; doi:10.5334/joc.521)
Supplement: Supplementary file 1 [file joc-9-1-521-s1.pdf]

Supplementary Material

To complement the primary analyses reported in the main text, we ran a mixed MANOVA on response times with RSI (500 ms vs. 5,000 ms), Stimulus (S1–S2, repeated vs. changed), and Response (R1–R2, repeated vs. changed) as within-participant factors and Memorization (memorizing vs. control) as a between-participants factor. Full results are reported in Table S1.

Table S1
Results of the Mixed MANOVA for Response Times

Predictor	df_{Num}	df_{Den}	F	p	η_p^2
Memorization	1	221	6.31	.01	.03
Stimulus	1	221	16.91	<.001	.07
Response	1	221	0.11	.74	<.001
RSI	1	221	81.69	<.001	.27
Memorization × Stimulus	1	221	6.57	.01	.03
Memorization × Response	1	221	0.51	.48	<.01
Memorization × RSI	1	221	0.15	.70	<.01
Stimulus × Response	1	221	126.59	<.001	.36
Stimulus × RSI	1	221	1.77	.19	<.01
Response × RSI	1	221	0.46	.50	<.01
Memorization × Stimulus × Response	1	221	28.71	<.001	.11
Memorization × Stimulus × RSI	1	221	0.09	.77	<.001
Memorization × Response × RSI	1	221	0.27	.60	<.01
Stimulus × Response × RSI	1	221	13.64	<.001	.06
Memorization × Stimulus × Response × RSI	1	221	0.30	.58	<.01

Note. 2 × 2 × 2 × 2 mixed MANOVA with RSI (500 ms vs. 5,000 ms), Stimulus (S1–S2, repeated vs. changed) and Response (R1–R2, repeated vs. changed) as independent within-participant variables and Memorization (memorizing vs. control) as a between-participants variable. df_{Num} indicates degrees of freedom numerator. df_{Den} indicates degrees of freedom denominator. η_p^2 indicates partial eta-squared. RSI = response-stimulus interval.

The corresponding mixed MANOVA on error rates, using the same factor structure, is reported in Table S2.

Table S2
Results of the Mixed MANOVA for Error Rates

Predictor	df_{Num}	df_{Den}	F	p	η_p^2
Memorization	1	221	0.09	.77	<.001
Stimulus	1	221	2.01	.16	.01
Response	1	221	3.98	.05	.02
RSI	1	221	50.11	<.001	.18
Memorization × Stimulus	1	221	0.00	.96	<.001
Memorization × Response	1	221	0.66	.42	<.01
Memorization × RSI	1	221	1.43	.23	.01
Stimulus × Response	1	221	94.79	<.001	.30

Predictor	df_{Num}	df_{Den}	F	p	η_p^2
Stimulus × RSI	1	221	5.00	.03	.02
Response × RSI	1	221	0.11	.73	<.01
Memorization × Stimulus × Response	1	221	9.87	<.01	.04
Memorization × Stimulus × RSI	1	221	0.12	.73	<.01
Memorization × Response × RSI	1	221	0.00	.95	<.001
Stimulus × Response × RSI	1	221	45.52	<.001	.17
Memorization × Stimulus × Response × RSI	1	221	4.50	.04	.02

Note. 2 × 2 × 2 × 2 mixed MANOVA with RSI (500 ms vs. 5,000 ms), Stimulus (S1–S2, repeated vs. changed) and Response (R1–R2, repeated vs. changed) as independent within-participant variables and Memorization (memorizing vs. control) as a between-participants variable. df_{Num} indicates degrees of freedom numerator. df_{Den} indicates degrees of freedom denominator. η_p^2 indicates partial eta-squared. RSI = response-stimulus interval.

Robustness of the Memory Accuracy–PRC Correlation to Outliers

The main text reports a positive correlation between S1 memory-check error rates and the difference in response-time PRCs between RSIs within the memorizing group (N = 109), r = .35, t(107) = 3.90, p < .001. To check whether this relationship was driven by a small number of extreme observations, we inspected the scatterplot of this association (Figure S1) and identified three participants as outliers: two with unusually large PRC differences between RSIs (approximately 1,000 ms and 500 ms, respectively) and one with an unusually high memory-check error rate (approximately 27%).

We re-examined the scatterplot with these three participants excluded (Figure S2, N = 106) and re-ran the correlation using both a Pearson and a rank-based Spearman test, the latter providing a check that does not depend on the influence of extreme values. Both tests confirmed a positive association of comparable magnitude to the original result, indicating that the correlation reported in the main text is not an artefact of outlier influence. Full results are reported in Table S3.

Figure S1

Scatterplot of S1 Memory-Check Error Rate and Response-Time PRC Difference, with Outliers Included

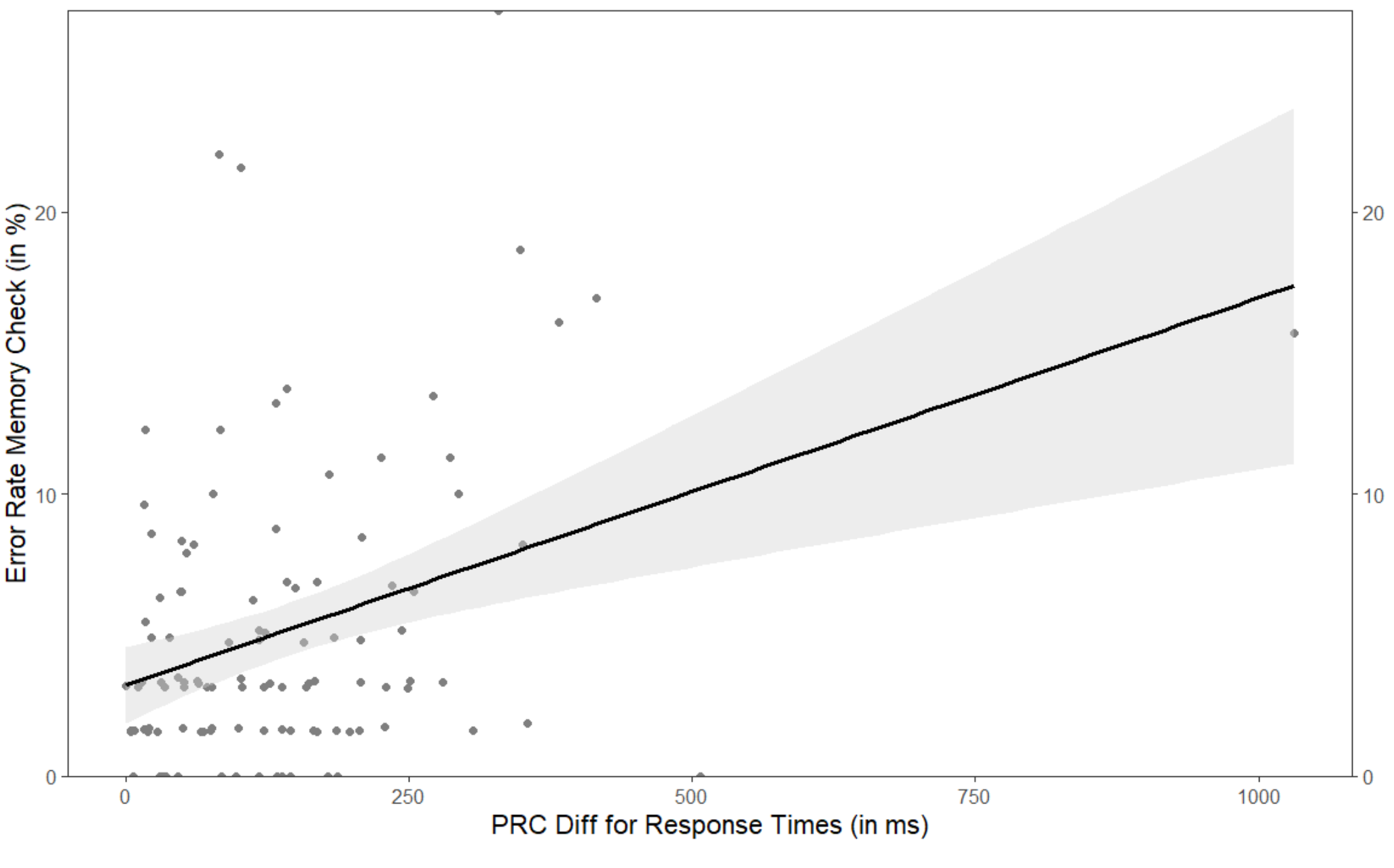

Note. N = 109. PRC Diff = difference in response-time partial repetition costs between short and long response-stimulus intervals.

Figure S2

Scatterplot of S1 Memory-Check Error Rate and Response-Time PRC Difference, with Outliers Excluded

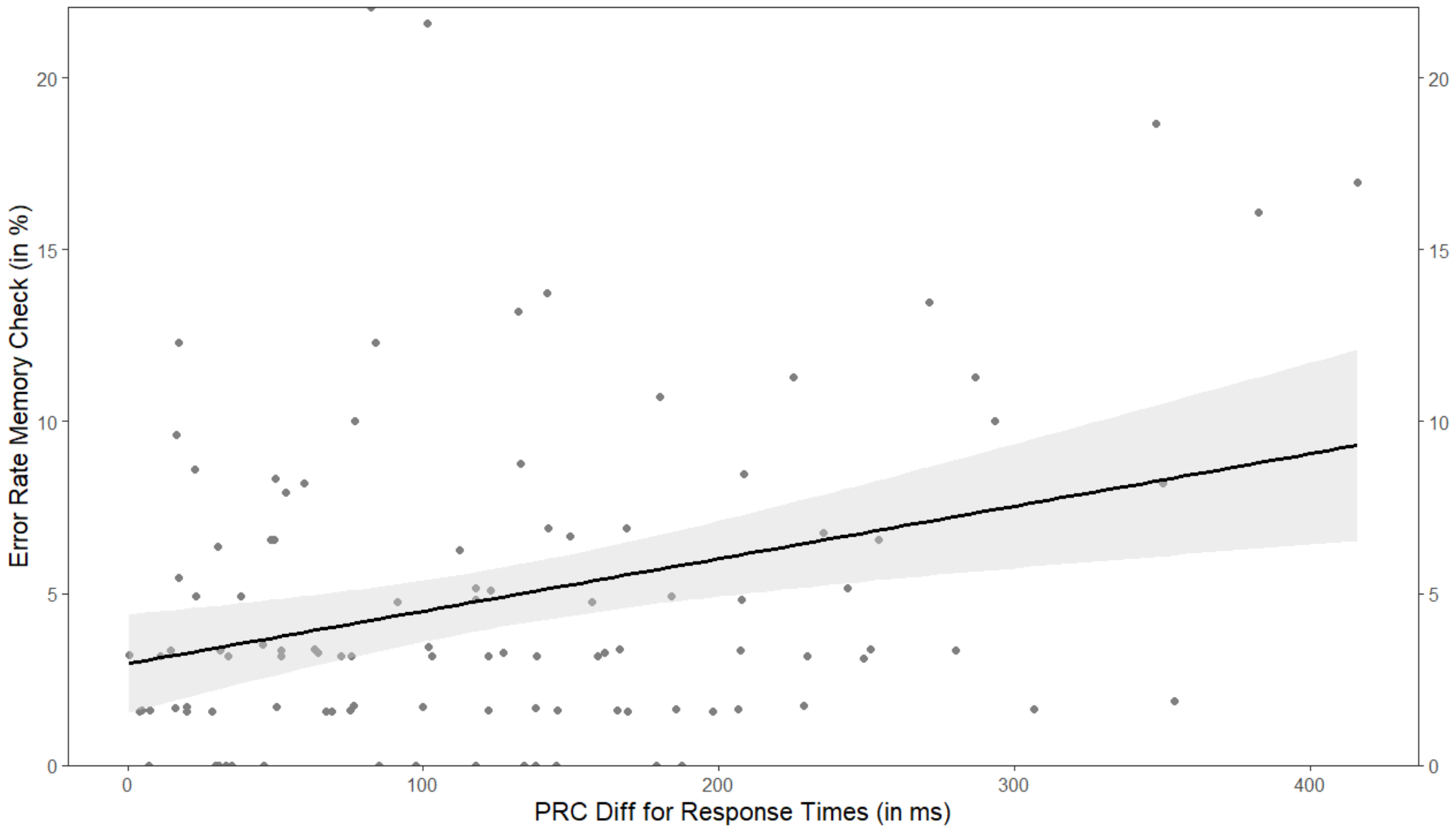

Note. N = 106. Three participants identified as outliers in Figure S1 were excluded (see text above). PRC Diff = difference in response-time partial repetition costs between short and long response-stimulus intervals.

Table S3

Correlation Between S1 Memory-Check Error Rate and Response-Time PRC Difference for the Memorization group, with and without Outliers

Sample	Test	Statistic	p	Estimate	95% CI
Full sample (N = 109)[1]	Pearson	$t(107) = 3.90$	< .001	$r = .35$	[.17, .50]
Outliers excluded (N = 106)	Pearson	$t(104) = 3.33$	.001	$r = .31$	[.13, .47]
Outliers excluded (N = 106)	Spearman	$S = 152{,}039$	.016	$\rho = .23$	—

Note. [1] As reported in the main text. All tests use S1 memory-check error rate and the difference in response-time PRCs between short and long response-stimulus intervals, within the memorizing group.

Memory-Check Accuracy Across the Experiment

Because the memory task drew on only two stimulus identities (pear vs. orange) repeated across the experiment, one might expect memory-check accuracy to decline over the course of the session as proactive interference from earlier trials accumulates. To evaluate this possibility, we plotted memory-check accuracy as a function of trial number, both overall (Figure S3) and separately for the short and long RSI conditions (Figures S4 and S5).

Contrary to a proactive-interference account, accuracy increased rather than declined across the session, rising from approximately 80–85% in the earliest trials to approximately 93–95% by the end, with a corresponding decline in error rates. This pattern was similar in both RSI conditions. This trend is more consistent with a practice effect in the memory-check procedure itself than with accumulating interference from the repeated use of the same two stimulus identities.

Figure S3

Memory-Check Accuracy as a Function of Trial Number, Overall

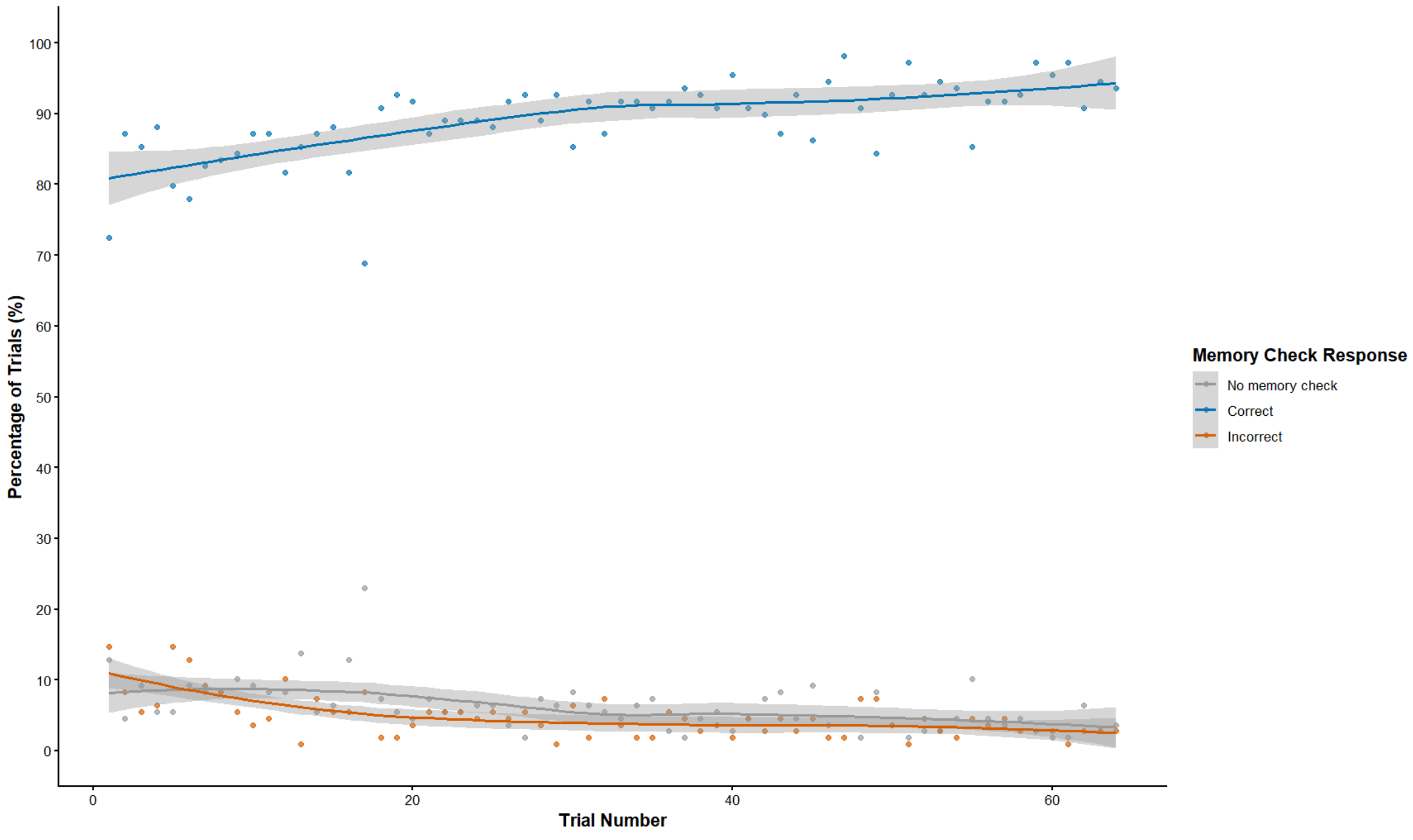

Note. Shaded bands represent 95% confidence intervals around the loess-smoothed trend.

Figure S4

Memory-Check Accuracy as a Function of Trial Number, Short RSI (500 ms) Trials Only

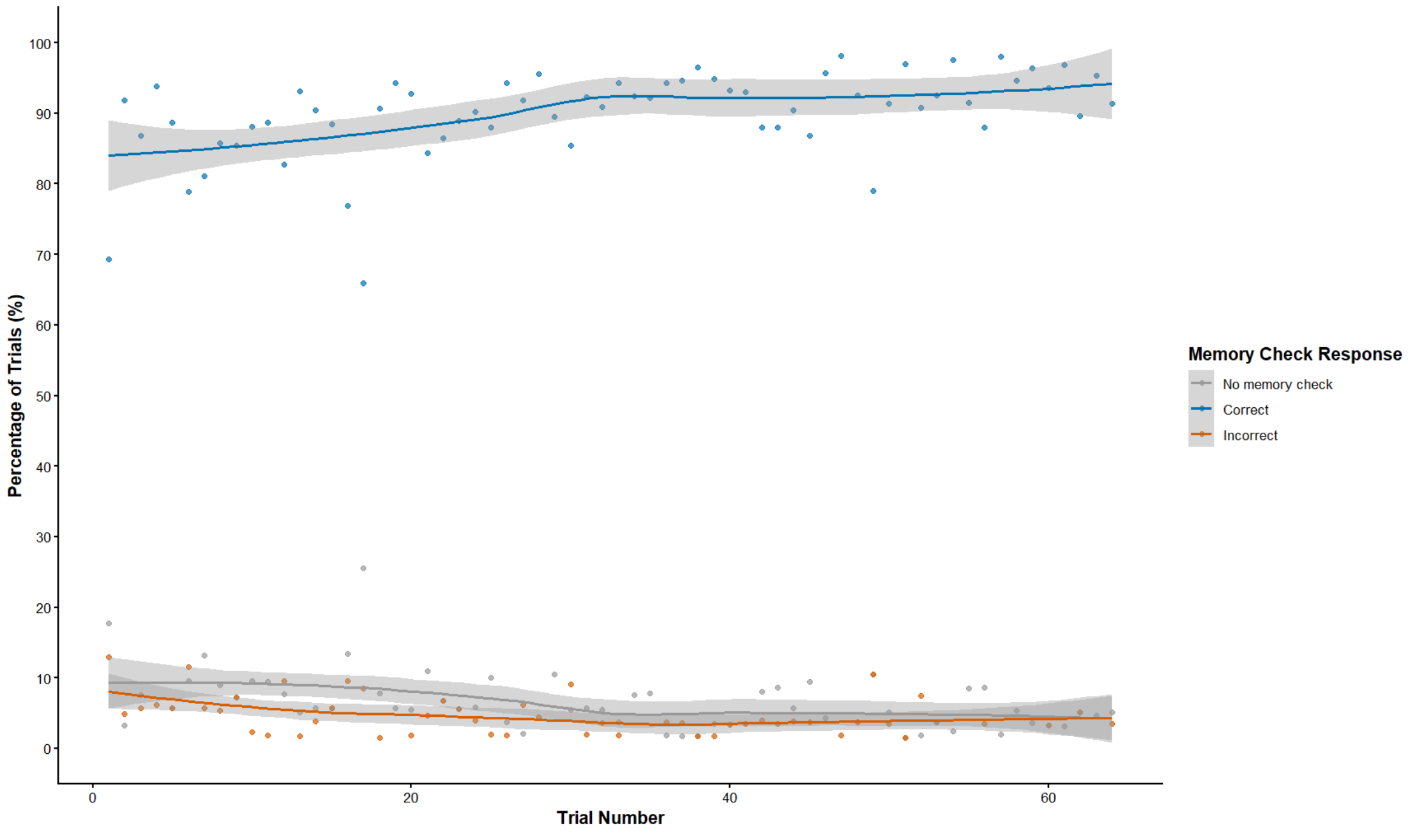

Note. Shaded bands represent 95% confidence intervals around the loess-smoothed trend.

Figure S5

Memory-Check Accuracy as a Function of Trial Number, Long RSI (5,000 ms) Trials Only

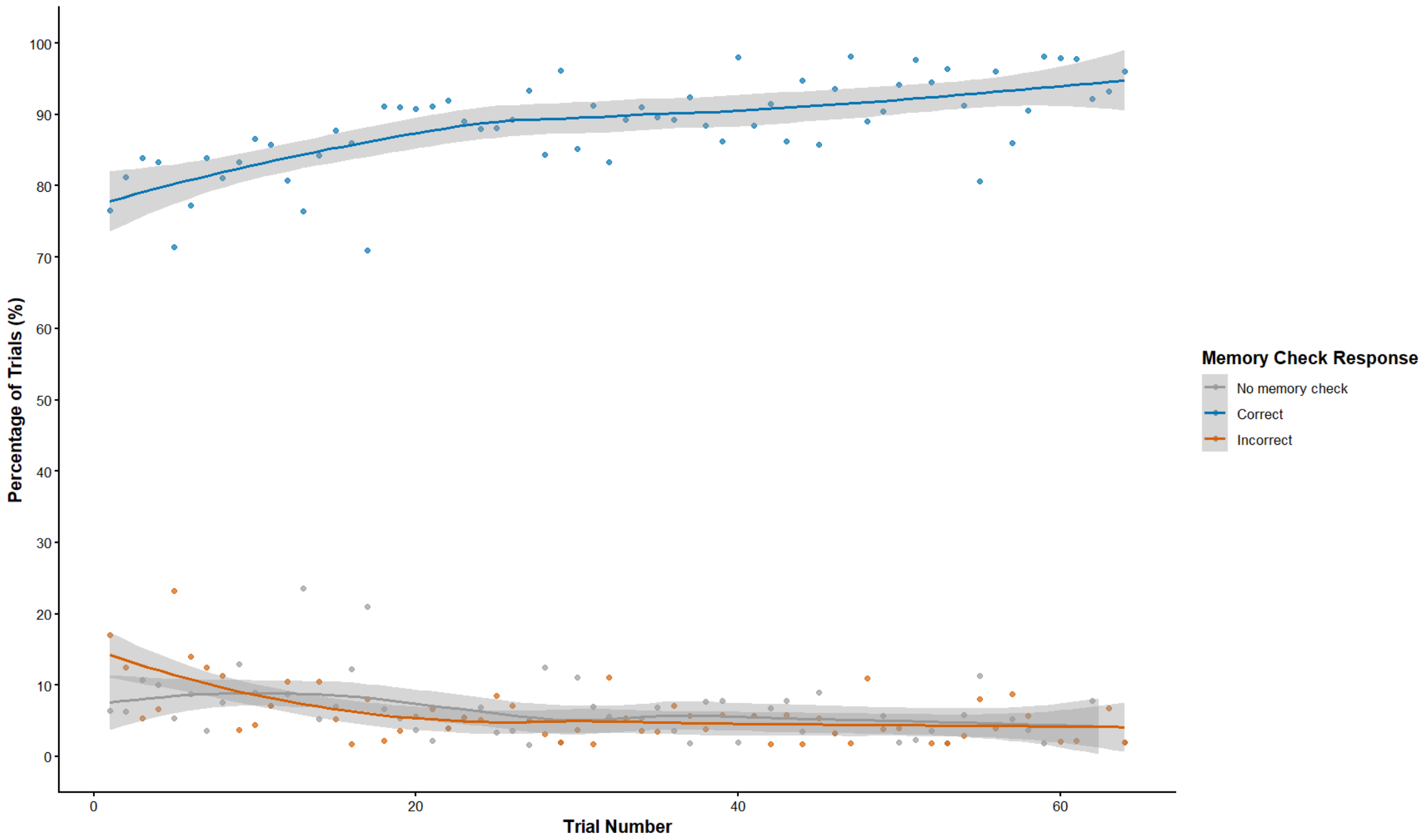

Note. Shaded bands represent 95% confidence intervals around the loess-smoothed trend.

Robustness of the Memorization × RSI Interaction to Trial Exclusion

To assess whether the RSI × Memorization interaction in error rates was driven by trials following an incorrect S1 memory check, we re-ran the primary MANOVA excluding these trials (4.33% of trials in the memorizing group; the control group is unaffected, as no memory checks were administered in that condition). Because each participant's per-condition means are now based on fewer trials, this restricted analysis has lower power to detect a true effect of the same size, independent of whether the effect itself is present. The shift from $p = .035$ to $p = .058$ should be read as a check on robustness rather than as evidence the effect is an artifact. Full results, alongside the corresponding response-time comparison, are reported in Table S4.

Table S4

Memorization × RSI Interaction, Full Sample vs. Excluding Incorrect-Memory-Check Trials

**Measure**	**Sample**	***F*(1, 221)**	***p***	η_p^2
Error rates	Including incorrect-memory-check trials	4.50	.035	.020
Error rates	Excluding incorrect-memory-check trials	3.64	.058	.016
Response times	Including incorrect-memory-check trials	0.30	.583	.001
Response times	Excluding incorrect-memory-check trials	0.13	.721	.001

Note. All values reflect the Memorization × RSI interaction term from the mixed MANOVA reported in Tables S1–S2 (full sample) and the corresponding re-analysis of the restricted sample.

PRC as a Function of Memory-Check Accuracy

To assess whether the RSI × Memorization interaction in error rates was driven by trials following an incorrect S1 memory check, we re-ran the primary MANOVA excluding these trials (4.33% of trials in the memorizing group; the control group is unaffected, as no memory checks were administered in that condition). Because each participant's per-condition means are now based on fewer trials, this restricted analysis has lower power to detect a true effect of the same size, independent of whether the effect itself is present: PRC variance in the memorization group was significantly larger than in the control group, $F(108, 113) = 1.67$, $p = .007$, *95% CI* [1.15, 2.43] (F-test for equality of variances), further reducing power to detect the interaction under the restricted sample. The shift from $p = .035$ to $p = .058$ should therefore not be interpreted as evidence the effect is an artifact. Full results, alongside the corresponding response-time comparison, are reported in Table S4.

Table S5

Participant Coverage for a Correct-vs-Incorrect Memory-Check PRC Comparison

Category	*n*
Total participants in memorizing group	109
With at least one incorrect-memory-check trial (either RSI)	88
With at least one incorrect-memory-check trial in both RSIs (minimum for a within-participant PRC comparison)	42
With full cell coverage (≥1 incorrect trial in each of the 4 Stimulus × Response conditions, at both RSIs) — the minimum for a valid PRC comparison	1

Note. Restricted to the memorizing group, within the final analysis sample. Each row applies a progressively stricter coverage requirement. Only the participant meeting the final row's criterion could in principle contribute a computable PRC to a correct-vs-incorrect comparison, which is far too few to support any reportable analysis.
